# Health Tract, No. 246

**Published:** 1866-06

**Authors:** 


HEALTH TRACT, No. 246.

### CELLARS.

There ought to be no cellars under any dwelling, because they are always more or less damp and musty; and are the receptacle of every variety of substances subject to decay, decomposition and the promotion of unheathful gases and odors; not one cellar in a thousand, either in town or country, is clean or dry; and as any housekeeper may verify in ten minutes, cellars are usually cluttered up with old barrels, boxes, casks, bottles, cast-off boots, shoes, hats; with bones, ashes, and various remnants of wilted and rotting potatoes, turnips, apples and other varieties of fruits and vegetables; it is the gases, the emanations, arising from these things, which cause the worst forms of typhoid and other malignant fevers. It is a benevolent arrangement of the wise and good Ruler of us all that pestiferous gases are lighter than the common air, and rise with great rapidity in warm weather to the regions of the clouds, where they can injure no one, and are either purified or resolved into their elementary conditions. Thus the disease engendering atmosphere of the cellar, rises upwards, penetrates the crevices of the flooring, and would escape from the building, but is confined to the parlors and chambers, especially on the highest floors. This is particularly the case in New York City, where the only entrance to the cellar is within the building, hence every time the cellar door is opened a crowd of foul emenations rush upward to impregnate the air of every apartment in the house. Very many of the ceilings of cellars are not even plastered; when really they ought not only to be plastered, but the eight or ten inches between the floors and the plastering should be filled in with charcoal or ashes. We have seen water closets under the stores in Broadway, which, for conditions of filthiness, are an utter disgrace to civilization. From considerations above named, the cellar should be the cleanest apartment in every dwelling; and in this moving time of the beautiful May, when perhaps half the dwellings change occupants, it is peculiarly convenient, when a cellar has been emptied by the movers out, for those moving in, to have the cellar most completely emptied of every thing not fast attached to the building; let every avenue of grating, door and window be left open day and night for at least a week; the floor, walls and ceilings or joists should be swept several times; the walls and ceilings whitewashed with two or three coats; the floor well washed and then rinsed with water, and unslacked lime or powdered charcoal should be liberally scattered wherever there is any appearance of dampness, so as to absorb all odors arising from moist and dark places. In a large district in a city the cholera appeared in only one house, traced to a pile of kitchen offal in a dark corner of the cellar.

